# Voluntary community service in medical school: a qualitative study on obstacles faced by student leaders and potential solutions

**DOI:** 10.3402/gha.v8.27562

**Published:** 2015-10-20

**Authors:** Alvona Zi Hui Loh, Julia Shi Yu Tan, Jeannette Jen-Mai Lee, Gerald Choon-Huat Koh

**Affiliations:** 1 Yong Loo Lin School of Medicine, National University of Singapore, Singapore; 2 Saw Swee Hock School of Public Health, National University of Singapore, Singapore

**Keywords:** voluntary community service (local, overseas), medical school, obstacles, solutions, mentorship, reflections, sustainability, global health

## Abstract

**Purpose:**

In medical school, students may participate in various community involvement projects (CIP), which serve disadvantaged communities. However, several obstacles may arise during these projects. The authors conducted a qualitative study with the primary aim of understanding the obstacles and corresponding potential solutions when medical students in Singapore participate in local CIP (LCIP) and overseas CIP (OCIP).

**Design:**

The authors recruited medical students from Yong Loo Lin School of Medicine, National University of Singapore, who were also leaders of a specific community service project done in medical school. Twelve one-to-one interviews were held for the participants from 6 to 8 January 2013. Participants were led in a discussion based on an interview guide. The interviews were audio-recorded and transcribed into free-flow text. Subsequently, content and thematic analyses of the transcripts were performed independently by three researchers.

**Results:**

The medical students faced many common obstacles during their community service projects. These obstacles include difficulties in recruiting and managing volunteers, attaining recognition or credibility for the project to acquire funding and resources, adjusting to a different culture or language, setting goals, and facing project-specific obstacles. Potential solutions were offered for some obstacles, such as building a strong executive committee for the project, grooming successive batches of leaders, and improving the project's public image, mentorship, reflections, and sustainability plans.

**Conclusions:**

Mentorship, reflections, and sustainability are potential solutions that have been proposed to tackle the obstacles faced during community service participation in medical school. However, there may still be difficulty in solving some of the problems even after these measures are put into practice. Future research may focus on evaluating the effectiveness of these suggested solutions.

Medicine is a service-oriented profession which requires professional attitudes and a sense of empathy and caring. Medical schools recognize the merits of community service for students and have incorporated it into their curriculum. Given that socio-cultural differences between the Asian and Western medical student exist, obstacles faced in community service by medical students in Singapore probably differ from that of the West. Singapore is an Asian country with underprivileged communities in both local and neighboring countries. The oldest medical school in Singapore, Yong Loo Lin School of Medicine (YLLSOM), offers a 5-year undergraduate medical education which does not incorporate community service into its official structured curriculum; therefore, all community service projects are student-initiated. Students entering this program are typically aged about 18–20 years.

Cashman and Seifer (1) present the numerous benefits of service learning during healthcare training, lending veracity to the increasing trend of community service. Even more so, community service in the field of medicine calls for a more rapid growth in maturity of the student in resolving obstacles and thinking out of the box to provide solutions in caring for the health of the communities around the region (2). In order to fine-tune the support given to medical students in their quest, this paper seeks to understand the obstacles that these students face, their corresponding solutions in the face of adversity, and how management strategies such as mentorship, reflections and sustainability plans are relevant in resolving their issues.

Indeed, more than one study has shown that community service participation among medical students is fraught with numerous challenges, but few have provided comprehensive means of resolving the issues. According to Holmes et al. (3), community service in a foreign location reports language as a main barrier, followed by challenges in the political situation, housing issues, and cultural adjustment. Yet, there is a lack in discussion of the solutions for the problems. These challenges may also include sustainability issues, some of which may be resolved with funding or innovation.

Reflections are undeniably integral to the improvement of a program, including community service. Many studies have supported the importance of reflections in learning and self-exploration (3–5). Additionally, reflections give an additional edge to improve inefficient or redundant processes in community service. Interestingly, a study by Welch (6) expounded on the ‘shadow-side’ of reflection, which could be interpreted as an unwanted consequence of reflections, offering an alternative view that reflections should be done strategically. Reflections have potential in crafting a community service project which maximizes help to the underprivileged when done appropriately, and in our paper we sought to explore the benefits and types of reflections.

Oftentimes, mentorship seemingly sparks confidence in students, but can be detrimental or useless if done without proper guidance. It usually comes in the form of either career-related sponsorship and coaching, or psychosocial role-modeling and acceptance (7). However, mentorship relationships may not be all positive as some mentors may abuse their power. Our study planned to further sieve out what constitutes a good mentor in service learning and how mentors can effectively guide the project without spoon-feeding the students.

In summary, the objectives of our study are as follows: 1) to identify the obstacles that medical students face when conducting community service projects, as well as the potential solutions to these obstacles, 2) to investigate how reflections can be used strategically to maximize its benefits when medical students conduct community service, 3) to find out how mentorship can be effectively used to guide medical students when they conduct community service, and 4) to explore how the medical students’ community service projects can be sustained.

## Methods

### Study design

We conducted a qualitative study using a phenomenological approach on the medical students of YLLSOM, each of whom was a leader of a specific community service project originating from the medical school. The phenomenological approach we adopted is described by Duffy (8), in which a qualitative study is used to study the empirical world from the perspectives of the subject, not the researcher. This implies that we aimed to understand the obstacles and potential solutions when conducting community service projects in medical school from the perspectives of the recruited subjects, that is, the community service project leaders, without influence by the researchers.

### Study setting

Twelve individual in-depth interviews were held for the participants from 6 to 8 January 2013 on the university premises. A set of study-specific topics were developed by GC-HK and AZHL to assess medical students’ experiences in community service in three domains: 1) nature of community service, 2) obstacles faced when participating in community service, and 3) potential solutions to overcome these obstacles to community service participation (Supplementary Table 1). For example, to assess the obstacles faced in community service participation, we asked, ‘What are some of the obstacles that you faced, when planning or conducting this community service project?’ The interviews were audio-recorded and transcribed into free-flow text. Subsequently, the transcripts were analyzed independently by three researchers.

### Participant recruitment

Only leaders of a community involvement project (CIP) were interviewed as we feel that they were in the best position to identify obstacles and solutions to their specific CIP, given their in-depth knowledge and experience gained from this role.

The inclusion criterion of participants is as follows: medical students from National University of Singapore (NUS) YLLSOM, who are leaders of community service projects done in medical school. The exclusion criterion of participants is as follows: medical students who fulfill the inclusion criteria, but are unwilling to participate in the study.

The NUS Medical Society, on behalf of the investigators of this study, disseminated an email to all medical students of YLLSOM, NUS, to invite the leaders of community service projects done in medical school to participate in our study, if they are willing to. The leaders of community service projects who were willing to participate responded by emailing one of the researchers (AZHL) in this study. Subsequently, interviews were arranged for and conducted with the participants.

### Data collection

All interviews were conducted in English by two of the researchers (AZHL and JSYT). Each interview was held in a comfortable, non-threatening atmosphere, lasting for approximately 30 min. The objective of the study was clearly explained to each participant and they were assured regarding the anonymity and confidentiality of their responses. It was also clearly explained that their participation was voluntary and they could withdraw from the session at any time. A free-flow discussion was allowed during the interview even though themes in the interview guide, which were derived from our literature review, were utilized to help facilitate the interview process. The leaders of the community service projects could elaborate on their experiences when conducting their respective projects. We were open to free discussion and the interview guide only served to prompt the interviewees about potential areas to discuss.

### Data preparation

All digital audio recordings made during the interviews were transcribed by JSYT. The responses were categorized into themes and sub-themes using context analysis.

### Data analysis

Three members of the research team (GC-HK, AZHL, and JSYT) independently reviewed and ratified the themes that had emerged. Thematic analysis was performed through a focus on identifying and describing both implicit and explicit ideas within the data (i.e. themes) that were derived from the interviews with our subjects, after audio-recorded interviews were transcribed into free-flow text. The content analyses were performed independently by three investigators in this study (9–15).

We followed the following steps: 1) data analysis plan by establishing analysis objectives, 2) data preparation through the creation of transcripts from the audio-recorded interviews, 3) reading and re-reading the notes and transcripts until we were intimately familiar with the content, 4) coding, by linking words or phrases in segments of the transcript to a common theme, 5) data display by writing displays to examine information on discrete topics, 6) data reduction by distilling information to extract the most essential concepts in the study, and 7) interpretation of data by identifying and explaining its core meaning which retains the perspectives of the subjects.

Themes were identified when the following elements were found in the transcripts: main point(s), repetition, local phrases/vernacular, metaphors and analogies, transitions, constant comparisons, and silence/missing data. Subsequently, we utilized content codes to organize our data, by coding themes that emerge in response to a question or from dynamic dialog. We adopted an exploratory approach by using codes which were content-driven and therefore derived from data. Themes and sub-themes were discovered and narrowed down to a manageable few, before hierarchies for themes were developed. The discrepancies in categorization were resolved by discussion. Thereafter, validation of the themes was carried out on the interviewees by member checking. Although we listed all quotations under various themes, we report those which best represented the views of the respondents for final interpretation.

Ethics approval was obtained from the NUS Institutional Review Board. Written informed consent was obtained from all participants.

## Results

### Nature of community service projects

Twelve in-depth interviews were conducted, with each community service project represented by a medical student who was project leader. There were four local CIP (LCIP) and eight overseas CIP (OCIP) leaders who were interviewed. The demographics of the LCIP and OCIP leaders, that is, their gender, age, year of study, how long they were involved in the project and how they became involved in the project, are listed in Supplementary Table 2. Medical students participate in community service projects which serve both local and overseas communities. Local communities include the financially disadvantaged, elderly residents, migrant workers in Singapore, palliative care patients. Overseas communities include slum dwellers, orphans, scavengers, and impoverished villagers in Cambodia, Myanmar, the Philippines, and Thailand. The nature of the medical students’ community service projects is presented in Supplementary Table 3.

From the interviews, Supplementary Table 4 shows an overview of the obstacles reported, solutions proposed, and how academics were affected as a result of the community service project. Table 1 summarizes the obstacles faced during CIP participation, while Supplementary Figure 1 shows the frequency of common obstacles being cited as a barrier by CIP leaders when they participate in community service project. Thereafter, Table 2 provides elaboration of proposed solutions to obstacles faced.

**Table 1:** Obstacles faced during CIP participation

Obstacles	Elaboration
Volunteer recruitment	Difficulty in finding sufficient doctors Difficulty in ensuring long-term volunteer commitment
Volunteer management	Difficulty in scheduling for volunteers to meet up to conduct the community service Difficulty in assigning roles and responsibilities to volunteers
Community service recognition	Difficulty in obtaining funding Difficulty in obtaining other resources
Cultural adjustment or language barriers	Difficulty in adjusting to different cultures and language especially in an overseas community service project Difficulty in understanding the mindset of the locals
Goal setting	Difficulty in setting the directions of the CIP project
Other project-specific obstacles	Difficulty in logistics management Difficulty in sourcing for permits to conduct CIP in foreign country Difficulty in ensuring safety of volunteers when conducting CIP Difficulty in helping volunteers attain self-sufficiency by supporting their own healthcare Difficulty in motivating the underprivileged to empower themselves

**Table 2:** Solutions to obstacles faced

Solutions	Sub-theme
Building a good committee	By reconstructing the structure of the committee By setting deadlines early
Grooming successive leaders	By motivating the juniors By creating handover documents
Improve the public's image of the project	By organizing publicity events
Mentorship	For mentorship: To give advice on how to start a project To help the team learn from past mistakes To provide specialized knowledge in a particular field To guide the project through other knowledge such as that of publicity and commercial practices To provide a final opinion, vet proposals, and propose new ideas for the team In the case where the mentor is a doctor: to serve as healthcare personnel in a clinical setting and provide medical expertise To help tailor the goals and directions of the CIP project To build trust with beneficiaries and/or external partners, by giving them a professional figure to speak to and liaise with Provide initial link-up to external organization and other contacts Against mentorship: The scale of CIP project is not extensive The project is not in need of professional expertise or guidance The CIP project has leaders and volunteers who are able to manage on their own by allocating specific roles and responsibilities The CIP project does not require a long-term mentor, but someone who can refer the team up to an external organization initially
Reflection	For reflection (on operations of community service project): To improve on the operations by reflecting on the ground situation and identifying the underlying problems in the project To fine-tune the program by sharing observations, re-evaluating the project's goals, and concluding on whether the beneficiaries’ needs were met To publicize the project by placing reflections on social media platforms For reflection (on individual growth): To allow for personal growth by reflecting on values learnt To allow for team bonding by sharing of personal thoughts, motivating each other, and creating a team with stronger bond Against reflection: The nature of the project was deemed to not require much reflection, such as a fund-raising activity Time constraints impeded the volunteers from reflecting
Sustainability plans	To plan for the future, such as implement a 5-year plan based on the present situation and anticipated future needs of the target population To learn from past events, through personal reflections for individual sustainability as well as improve systems for more efficient output To have proper leadership succession, such as by having the juniors shadow the seniors, creating handover documents to recount the past problems and they can be better tackled by future committees, and creating a project-specific leadership training program To ensure self-sufficiency among the beneficiaries, e.g., by involving a sustainable source of help such as that of the government. To have dedicated past volunteers who return and offer their services, to inspire the team as well as offer medical expertise if they have graduated as doctors To secure a constant source of funding or recuperation of funds To incorporate technological advancements into their plans, as technology may help to reach the target beneficiaries faster and more efficiently

### Obstacles faced during CIP participation

Six main obstacles were faced by the student leaders in their community service project: 1) recruiting volunteers and ensuring their commitment to the CIP project, 2) managing volunteers in terms of scheduling, assigning roles and responsibilities, 3) obtaining recognition for CIP project and subsequently its funding and resources, 4) adjusting to a different culture or language where the CIP project is conducted, 5) setting the future direction of CIP project, and 6) other project-specific obstacles (Table 1).

#### Volunteer recruitment and ensuring commitment

The issue of recruiting volunteers and keeping them committed was common to many groups.

One of the problems we meet every year is to find sufficient doctors to come on the trip with us. (Project Battambang)

#### Management of volunteers

A subsequent problem is the management of the volunteers, such as assigning roles and responsibilities to each of them, motivating them, allocating sufficient time for each of them to do their work and overseeing the needs of each person.

There are many aspects to consider, to think ahead, further than your team to see how everyone is working at a comfortable pace. (Project Sa'bai)

#### Obtaining recognition, funding, and resources

The majority of student leaders also highlighted the difficulty in obtaining funds for their project, with some mentioning that this could be due to a lack of recognition of the project.

There is the issue of money. We couldn't get any sponsors until later on. (Project Happy Apples)

#### Cultural adjustment

Many students involved in OCIP faced difficulty in adjusting to the culture and language of the foreign country.

The main obstacle was communication with the local NGO. I forgot that the culture there is very different from the culture here. (Payatas Medical Outreach)

#### Determining future direction of the CIP

The students also found difficulty in setting goals, as they had to reach a compromise between the aims of their community service project and the requirements of their sponsoring company or organization. Another reason for this particular difficulty was uncertainty in the benefits of their service.

The Eagle sponsorship wanted us to help more people, which we cannot promise because we go back to the same communities. There was a clashing of directions. (Manila Medical Mission)

#### Other project-specific obstacles

Each community service group faced difficulties specific to their own project, including safety issues and implementation of new ideas.

We go to rural areas and there is no one around to ensure our safety. (Manila Medical Mission)

### Potential solutions to overcome obstacles to community service

Possible solutions were offered for some of the obstacles, such as 1) building a strong executive committee, 2) grooming the successive batches of leaders, 3) improving the project's public image, 4) mentorship, 5) reflections, and 6) sustainability plans (Table 2).

#### Building a strong executive committee

Building a strong executive committee by maintaining a structure of the CIP leaders was proposed as a potential solution to overcome obstacles during CIP participation, especially when the previous system was deficient in structure and leadership.

Usually there is an executive committee, but because we are a very small group, we didn't have any structure. (Operation Smile Student Chapter)

#### Grooming successive batches of leaders

Grooming the successive batches of leaders so that there is a proper handover of roles and responsibilities is another potential solution to overcome obstacles in the long term.

During the handover period, I ensure my committee knows how their roles and responsibilities can be handed over. (Project Happy Apples)

#### Improving the project's public image

Another potential solution is to improve the project's public image through greater outreach, which may help to increase funding, resources and external partners, which are at the core of every CIP project.

Publicity is important for the project to survive over many years, because that's where the source of your revenue and resources come from. (Project Sothea)

#### Mentorship

Mentorship was clearly evident in some CIP projects, while lacking in others. Six CIP projects found mentorship helpful in offering advice, serving as a familiar bridge between the existing locals and the changing committee and volunteers, as well as providing the contacts necessary to engage the partners for the project. However, the other six leaders interviewed found that mentors were not necessary for the project due to the scale of the project and the high levels of maturity of leaders and volunteers.

Students felt that their mentors offered them invaluable advice due to their long-standing experience, and they provided a longer term perspective on the project which enabled them to ensure sustainability and avoid repeated mistakes. A student remarked that a mentor would be required if he or she is specialized in a particular field and had access to knowledge that was otherwise less accessible to a medical student.

Mentors help us by advising our project to make sure that we are not repeating any mistakes from the past. (Neighbourhood Health Screening)

He (our mentor) gives us the liberty to do what we want, but also gives good advice on some things that we won't be able to decide on. (Project Battambang)

The mentor also acted as a familiar face, for the locals to identify the service group with.

Mentors are definitely useful as they give the locals a face to attach themselves to. (Project Lokun)

Furthermore, mentors could offer contacts and link the group to a larger or supporting organization to ensure the smooth running of the project, as well as access to funding and resources.

He (our mentor) is definitely useful for his contacts. (Project Sothea)

Yet, some students found that having a mentor is not necessary and that the group can cope up with the responsibilities required of them.

We are a small interest group, not a very extensive project, so we are fine doing it on our own. (Operation Smile Student Chapter)

#### Reflection

Ten community service groups carry out reflections; one does not; and one of the projects has two groups of people, one of which carry out reflections and the other does not. Some students found reflections helpful in improving operations, enhancing personal growth and team bonding, and promoting publicity. However, a few students did not do reflections as they felt there was nothing to reflect or there is insufficient time.

Reflections improved operations as there is constant evaluation, and the group strives to do more and better for the target beneficiaries. Additionally, it aids in tailoring the program for future sustainability.

(Reflection is) useful for processing everything you have experienced. (Project Sa'bai)

Many students testified that personal growth was enhanced through reflections.

I think that reflection is definitely necessary, it personally sustains you, and you need to think about what you did and how you can improve it. (Project Lokun)

By sharing one's thoughts and sentiments during reflections, the team bonded and understood more about one another.

The reflection sessions are always very thought-provoking. We reflect on values, our perspectives on things, which are really personal. (Project Battambang)

The reflections also helped to garner publicity when posted on social media platforms.

The volunteers write reflections which are publicized on our Facebook page if they agree to it. This helps people know what we are doing on the ground. (Project Happy Apples)

However, some students felt that there was nothing to reflect on, as the volunteer work did not involve a lot of self-assessment.

It (reflection) is based on student interest. We raise money, in which there is not much reflection to do. (Operation Smile Student Chapter)

Also, a student felt that there was not enough time for reflections during the OCIP.

The reflection session became a logistics session, everyone was so tired during that time. (Project Yangon)

### Sustainability plans

In terms of ensuring sustainability, several options were proposed: to plan for the future, learn from past events, have proper leadership succession, ensure self-sufficiency among the beneficiaries, have dedicated past volunteers who return and offer their services, secure a constant source of funding or recuperation of funds, and incorporate technological advancements into their projects.

The students find that planning for the future and reflections about the past events helped in facilitating the implementation of the project.

We always place a strong emphasis on sustainability. We try to project a few years in advance. (Neighbourhood Health Screening)

We keep the project sustainable through constant reflection and thinking about whether our project is working. (Project Battambang)

Also, leadership succession helped the student leaders ensure that successive batches were well trained for the CIP to be handed down. Additionally, the dedication of previous volunteers was thought to sustain the project when they returned and offered their services.

We have leadership succession, in which juniors take over the project and lead the project. (Project Sa'bai)

While some groups experienced a shortage of funds, other student leaders ensured that the costs for the project were recuperated so that the project was self-sustaining. Other groups found a constant source of funding.

There are no costs in terms of manpower, and the rental is free, so it is very sustainable and cost-effective. (Constructing Care Collaboration)

We cannot keep on fundraising forever. Thankfully, XXX (an organization) has taken over in terms of the funds, so we do not need to worry about money again. (Payatas Medical Outreach)

Some community service groups intend to help out in other way, even if long-term sustainability could not be assured. Other groups sought to achieve sustainability through educating the locals.

We primarily educate the patient through prevention of disease. Examples such as hygiene and eating clean food will definitely improve their health in the long term. (Project Sothea)

One particular project found it easier for the government to support its programs and ensure future sustenance. Most OCIPs have grasped the importance of ensuring the empowerment of the locals to help themselves, and their sustainability plans include the involvement of the locals themselves (16).

Payatas has the criteria for sustainability. Medical treatment for tuberculosis is free in Philippines, so we do not need to constantly think about raising funds. (Payatas Medical Outreach)

Innovation also had a part to play as technology help to reach the target beneficiaries faster and in more efficient ways.

We tried to implement the iPad at NHS, where we are very focused on getting information from our residents. We used paper forms before, but an iPad app is in the works to collect data, collate it and merge it for us. (Neighbourhood Health Screening)

## Discussion

### Proposal of solutions to obstacles faced

To date, few studies propose solutions to the obstacles faced during the participation of community service. Holmes et al. (3) highlighted four aspects of obstacles to global health electives: language, challenges in political situation, housing issues, and cultural adjustment. McCarthy et al. (17) also highlighted obstacles similar to those we identified, further affirming the recurring themes of obstacles in service learning: cultural differences inducing scepticism among locals, communication issues, conflicting objectives among sponsors, the team and the hosts, and to a lesser extent in our study, expectations of the hosts.

The nature of the community service projects, including the location where it was conducted, has some bearing on the obstacles encountered when participating in the projects. An obstacle in point is the difficulty in attaining the sustainability of a CIP in less developed countries, such as through fostering responsibility for project sustainability by the people in the home country. It is not easy to help those volunteers attain self-sufficiency in their country in terms of healthcare. In contrast, a more developed country would face this problem less due to the greater wealth, resources and education of the people, allowing them more time, energy to contribute to society. However, there are also general obstacles involved in the process of conducting any project whether it is local or overseas, such as the recruitment of volunteers, the maintenance of the commitment levels, the procurement of funds, which arises from the increasingly hectic lifestyles of medical students and the inherent difficulty in obtaining cash.

In our study, the students suggested a few solutions to certain issues, such as building a strong executive committee for the project, grooming the successive batches of leaders, and improving the project's public image. Notably, mentorship and reflection time may potentially solve many of the obstacles encountered.

### Utility of reflections and mentorship

Reflections are undeniably integral to the improvement of a program, including community service, which confirms the findings of other studies which support reflections in learning and self-exploration (3–5, 17, 18). We recognize that Welch (6) has proposed unwanted consequences of reflections, offering an alternative view that reflections should be done strategically as they may worsen the emotional and cognitive tensions that occur during these experiences. However, the students we interviewed did not mention any of these downsides to reflections. In contrast, we found that the majority of students felt that reflections were helpful in team bonding, improving in operations, tailoring of the program, and promoting personal growth. The positive effects of reflections that we found are in line with Beylefeld's (18) findings that reflections can help undergraduate medical students integrate their understanding gained from experiences. However, his study also emphasized the importance of clear guidelines in reflective learning, which have yet to be developed for medical students who have undergone community service in medical school. We propose that future studies focus on the specific areas and sequential methods during reflections, so that a set of guidelines can be developed and utilized for reflective learning in community service done in medical school, for the benefit of medical students. In addition, a unique and currently unexplored benefit of reflections is that it could also bring publicity to the project, when students provide testimonies of their experience on social media platforms.

Although group reflections would require students to honestly discuss their feelings and opinions to one another, there had not been any observable negative consequence, which was also pointed out by Welch (6). However, some students hold the view that some CIP projects do not require reflections, in the event of shortage of time or when there is nothing to reflect about, such as in fund-raising events. Reflections have potential in crafting a community service project which maximizes help to the underprivileged when done appropriately.

Similar to the study by Weinreich (7), we found that mentorship can guide in the dispensing of knowledge and advice, and in providing contacts for relevant organizations and people. McCarthy et al. (17) also stated that a mentor was helpful in managing the expectations and scope of the participants. Interestingly, our study found that mentors may act as a familiar figure for the underprivileged to identify the service group with, as the students who head the team may change on a regular basis. On the other hand, some students claimed that their project do not require mentors due to its small scale, and the ability of volunteers to carry out their duties well without the need for mentorship. This may help to improve their leadership skills in terms of ability in fund-raising, volunteer recruitment, managing the volunteers, setting goals, and motivating others, which is congruent with the findings of Goldstein et al. (19).

We note the strong, growing demand from medical students and the changing societal forces that call for better global health training (20), and that there has been a burgeoning interest in global health teaching in undergraduate medical curricula (21). In view of these, we propose that the results of our study may be used to develop the global health programs in medical schools by identifying common obstacles to the local and overseas community service, and guiding students in overcoming them.

### Identification of areas for sustainability

Notably, we have identified the following areas for sustainability: advanced planning, periodic reflections, training successive batches, recuperation of costs, encouraging dedication of previous volunteers, funding, government help, education of the target population, and innovation. Although language, cultural, and political issues were briefly mentioned, new obstacles to CIP participation in medical school were related to the leadership aspects of the project, such as recruiting volunteers and keeping them committed, management of people and time, setting goals, obtaining the funds and resources required to sustain the program, as well as some project-specific obstacles. As our study was conducted on community service leaders, we believe that this could be one of the reasons which explain why the obstacles with relation to leadership issues were more apparent.

Brush et al. (22) described that in Tulane University School of Medicine, a faculty member who serves as a part-time Director of Service Learning assists students with developing sustainable projects, integrating activities with course learning objectives, providing institutional administrative oversight, and securing resources for projects. This is linked to their course objectives and student learning outcomes through required reflection questions specific to each service project and developed by the service-learning leader with faculty guidance. However, the crucial difference between the community service of their student population and ours is that in their university, community service is compulsory in a structured program with course credits, which differs from that in our student population, which is voluntary without course credits. Therefore, we postulate that the aims, nature and student experiences during the conducting of community service projects would be vastly different. We believe that we can adopt a modified model, where instead of having one faculty member serve as a mentor for all projects, different mentors with the relevant expertise for each community service project can be appropriately matched to them. This ensures that the mentor has the specific knowledge and contacts to assist the students in their projects, which targets a myriad of beneficiaries of different demographics and needs, and who are located in different countries.

### Limited power due to small sample size

We acknowledge that this is a qualitative, single-centered study which reports only 12 interviews which limits the power of the study. We had 12 interviewees in our qualitative study, which some may deem as a small number. According to Sandelowski (23), inadequate sample sizes can undermine the credibility of research findings. However, we note that no computations or power analyses can be done in qualitative research to determine *a priori* the minimum number and kinds of sampling units required (23). We believe that our qualitative study, despite having limited power due to small sample size, may have this limitation compensated for by the quality of response from the participants. Patton (24) states that the ‘logic and power’ of various kinds of purposeful sampling used in qualitative research lie primarily in the quality of information obtained per sampling unit, as opposed to their number *per se*. In addition, an aesthetic thrust of sampling in qualitative research is that small is beautiful (25). Therefore, we hope that despite having a small sample size, the considerably high quality of our discussion as evidenced by the insightful responses from our interviewees who had much to offer and share during the interviews will offset the limited power in the study.

### Potential self-selection bias

Only medical student leaders who responded to the invitation to this study and took out time to participate in our interviews were included in the study. Therefore, potential self-selection bias (26) might have favored those who were interested in the subject of community service as well as the research on it, and those who had time to join the interviews.

### Researcher bias

We also recognize the potential issues of researcher bias in our study, when the researchers’ preconceptions, motivations, and ways of seeing shape the qualitative research process. However, there is ample evidence to suggest that researcher's motivations and preconceptions shape all research (27). To minimize this problem, three investigators had to analyze the data independently. Subsequently, the researchers synthesized their analyses before starting the manuscript writing. By having more than one investigator analyzing the data obtained, we aim to prevent a single researcher's preconceived notions from creating a result that is skewed toward a particular researcher's ideas which could be unrepresentative of the study participants’ perceptions. One perspective on the other hand is that if researcher subjectivity could not be completely eliminated, it can be viewed as ‘something used actively and creatively through the research process’ rather than as a problem of bias (28).

### Reliability

The qualitative nature of our research is considered less reliable than that of quantitative investigation. The reliability is weakened as the process of qualitative studies is under-standardized and relies on the insights and ability of the observer (8). However, the concept of validity requires understanding the beliefs about the nature of reality (29). The belief that there is one observable reality knowable through the process of research, albeit sometimes imperfectly, is typically associated with a positivist paradigm in quantitative research, but also with the realist paradigm in some qualitative research (30–32). Such qualitative research strives for truth through the qualitative research process, for example, by having outside auditors or research participants validate findings (29). Even though we did not have an auditor validate our findings, one of our investigators who was not involved in thematic analysis, performed member checking to give input on our complied data and to provide a balanced viewpoint on the adequacy and accuracy of our data. We adopted the view of reality emerging from an interpretivist perspective and aimed to depict the truth as well as we possibly could.

## Conclusions

In our study, we have identified the obstacles that medical student leaders face when conducting community service, namely difficulties in recruiting and managing volunteers, attaining recognition or credibility for the project to acquire funding and resources, adjusting to a different culture or language, setting goals, and facing project-specific obstacles. This paper also explored the proposed solutions to the obstacles such as mentorship and reflections, as the students largely found that they produced a positive outcome. Although various potential solutions have been proposed to tackle the obstacles faced, there may still be difficulty in solving some of the problems even after these measures are put into practice. Future research may focus on evaluating the effectiveness of these suggested solutions in real life, after the proposed solutions have been implemented.

We hope our findings will be used to help facilitate community service participation among medical students, assist students in overcoming the challenges that occur during community service, and enhance the professional development of medical students in Singapore.

## Supplementary Material

Voluntary community service in medical school: a qualitative study on obstacles faced by student leaders and potential solutions
