# Supplementary material for: Voluntary community service in medical school: a qualitative study on obstacles faced by student leaders and potential solutions
Source: Glob Health Action. 2015 Oct 20;8:10.3402/gha.v8.27562. doi: 10.3402/gha.v8.27562 (PMC4613900; doi:10.3402/gha.v8.27562)
Supplement: Voluntary community service in medical school: a qualitative study on obstacles faced by student leaders and potential solutions [file GHA-8-27562-s001.docx]

**Appendix (Supplementary Material)**

**Table 1.** Qualitative interview guide

| **Nature of community service projects** | - How did this Community Service Project begin? - Is this Community Service Project open to only medical students, or other health-related faculties as well? - How were students recruited into this Community Service Project? Was there a selection process (e.g. submission of write-up/ interview)? - How many volunteers are in this Community Service Project? - What do volunteers in this Community Service Project do? - Who is the target population of the Community Service Project? - When and where is this Community Service Project conducted? - How do you think this Community Service Project has benefitted the target population? - Is this Community Service Project one-time event? If not, how does the team keep this Community Service Project sustainable? - Is there a Teacher-in-charge or Supervisor for this Community Service Project? If so, what is his/ her role, and was he/ she helpful in guiding the team? - Is there a reflection time (in speech/ writing, individually or as a group) for the Community Service Project after completion? |
| --- | --- |
| **Obstacles faced when participating in community service** | - What are some of the obstacles that you faced, when planning or conducting this Community Service Project? - Was your academic work affected, whether positively or negatively, due to your participation in this Community Service Project? |
| **Potential solutions** | - What do you think are some improvements that can be made to this Community Service Project to overcome these obstacles? |

**Table 2.** Demographics of the LCIP and OCIP leaders

| **Name of project** | **Project leader's gender, age (in years), year in medical school** | **How long they were involved** | **How they became involved** |
| --- | --- | --- | --- |
| **Operation Smile Student Chapter** | Female, 21, Year 2 | 4 years | Joined and took leadership of the pre-existing project in junior college (i.e. pre-college) out of interest, and decided to introduce the project into medical school and continue the leadership |
| **Neighbourhood Health Screening (NHS)** | Male, 20, Year 2 | 2 years | Joined the pre-existing project in university out of interest, and took leadership of the project after being involved for some time |
| **Project Happy Apples** | Male, 21, Year 3 | 3 years | Started up and took leadership of the project in university out of interest of the target group of beneficiaries (i.e. palliative care patients) |
| **Constructing Care Collaboration (CCC)** | Male, 21, Year 3 | 3 years | Started up and took leadership of the project in university out of interest of the target group of beneficiaries (i.e. migrant workers in Singapore) |
| **Project Sa’Bai** | Female, 20, Year 2 | 2 years | Joined the pre-existing project in university out of interest, and took leadership of the project after being involved for some time |
| **Project Yangon** | Female, 20, Year 2 | 1 year | Started up and took leadership of the project in university out of interest of the target group of beneficiaries (i.e. needy people in Yangon, where the leader's home country is) |
| **Manila Medical Mission** | Female, 20, Year 2 | 2 years | Joined the pre-existing project in university out of interest, and took leadership of the project after being involved for some time |
| **Project Lokun** | Male, 20, Year 2 | 2 years | Joined the pre-existing project in university out of interest, and took leadership of the project after being involved for some time |
| **Project Khon Kaen** | Male, 22, Year 4 | 4 years | Joined the pre-existing project in university out of interest, and took leadership of the project after being involved for some time |
| **Payatas Medical Outreach** | Female, 22, Year 3 | 3 years | Joined the pre-existing project in university out of interest, and took leadership of the project after being involved for some time |
| **Project Battambang** | Female, 20, Year 2 | 2 years | Joined the pre-existing project in university out of interest, and took leadership of the project after being involved for some time |
| **Project Sothea** | Female, 20, Year 2 | 2 years | Joined the pre-existing project in university out of interest, and took leadership of the project after being involved for some time |

**Table 3.** Nature of community service projects in medical school

| **Name of project** | **Location of project** | **Environment in which project was conducted** | **Type of work in community service project** | **Profile of volunteers in community service project** | **Method(s) of volunteer selection/ recruitment** | **Frequency of project** | **Target beneficiaries** | **Type of reflection done, if any** | **Methods for fund-raising/ resource-raising** | **External partners of community service project** |
| --- | --- | --- | --- | --- | --- | --- | --- | --- | --- | --- |
| Operation Smile Student Chapter | Local, i.e. Singapore | Local schools when fundraising  Local hospitals when interacting with the target beneficiaries | To raise awareness for Operation Smile  To raise funds for Operation Smile | 30 second-year medical students and over 20 first year medical students | Announcement to recruit interested volunteers | No specified frequency, as the project is events-based | Children born with cleft lips in Singapore and other countries | No reflection done | Shirt sale and Valentines’ Day Sale. | Operation Smile International,  KK Women’s and Children’s Hospital |
| Neighbourhood Health Screening (NHS) | Local, i.e. Singapore | Neighbourhoods such as MacPherson, Tanjong Pagar, Taman Jurong and Eunos Crescent. A central location such as a void deck is designated on top of the door-to-door visits to target beneficiaries. | To provide health screening and follow-up services for poor elderly in the neighbourhood, specifically monitoring of blood glucose, blood pressure, hyperlipidemia, cancer and lifestyle habits. | First type is committee members (Medical students only), 20 M1, 2 M2. Second type is those who participate in screenings: medical students, nursing students, social work students, dentistry students, 150 per day for 4 days. | First type: announcement, selection and interviews.  Second type: announcement and sign ups only, with no selection process | Screenings are conducted once a year around August to September. Follow-ups are projected to be once every three months. | Elderly residents who face financial difficulties. | None for volunteers on the ground, but debrief/ reflections for committee members during General Meetings | Financial resources from Dean’s Office and grassroots/ community centres. | Social Work Organisations like Family Service Centres, Code 4, polyclinics who agree to do follow-up, Tzu Chi, grassroots or community centres like Marine Parade GRC, or Jurong GRC, Dean’s Office. |
| Project Happy Apples | Local, i.e. Singapore | Predominantly Bright Vision Hospital, a community hospital with palliative care inpatient service. | Initially, to sell apples to raise funds for palliative care patients. Currently, to raise awareness on palliative care patients by interviewing them and using creative media (posters, publicity video, presentation) to publish their stories.  To carry out events in Bright Vision Hospital monthly and befrienders sessions weekly. | Over 40 volunteers from medicine and allied Health faculty i.e. nursing, pharmacy. | Announcement to recruit interested volunteers | For the community outreach, no specified frequency. | General Public (raising awareness).  Palliative care patients (psychosocial support). | Handover documents where volunteers recount the obstacles they have faced for future generations. Volunteers write reflections which would be publicized on our Facebook page. | Sponsorship from Dean’s Office for printing. | Singapore Hospice Council. |
| Constructing Care Collaboration (CCC) | Local, i.e. Singapore | Community clinics located in Foo Chow Methodist church and a Jurong dormitory. | To help out at the community clinics catered to migrant workers, help the doctors there, as well as interact with the migrant workers.  To organise events like public seminars to discuss issues faced by migrant workers, skin seminar to discuss chronic illnesses that workers contract | 2 cycles of medical students. Each cycle has about 56 medical students, and lasts 6 months long. | Announcement to recruit interested volunteers, held a survey online but accepted almost everyone who applied | Each volunteer goes down to a clinic once a month, 6 to 7 volunteers in total will rotate by joining the clinics once a week. | Singapore’s migrant workers. | At the end of each session, 15-30 minutes group reflection guided by the handbook by Prof Hooi Shing Chuan about the various aspects of the clinic and the patients. | NIL. | HealthServe, a Christian doctor network. |
| Project Sa’Bai | Overseas, central Phnom Penh, Cambodia. | Initially held clinics in Don Bosco schools (schools under the Salesian Sisters of Don Bosco), then expanded to village clinics and kampongs. | To participate in clinics and educate children from Grade 1-7/8 about general hygiene and puberty  To build water filters for target beneficiaries  To work with partners build infrastructure like schools | 30 medical students (20 M1s and 10 M2s). Other volunteers include dental students, doctors, nurses, and nursing students from polytechnics | Year 1s fill up an application form to select 2 of the 4 existing Cambodian OCIPs in medical school, and are selected based on individual interviews. | Once a year. | Poor living in slums of Phnom Penh, 3 Don Bosco schools and 2 villages. For school children, mostly elementary, including kindergarten and older, average about 300 kids. Village clinics see 300 patients per day as well, | Reflection session in small groups and a sharing on the last day of the trip. | Fund-raising activities, or donations from willing donors, write emails to organizations related to children or Cambodia for sponsor in cash or in kind like equipment or drugs. | ACTS, A Call To Share. KK Hospital. |
| Project Yangon | Overseas, Yangon, Myanmar. | Orphanages in villages. | Initially, to provide health screening and teach personal hygiene at ten orphanages  Currently, to supplement health clinics in the outskirts of Yangon and bring health education to the villagers | 14 medical students | Recruitment drive for M1s and M2s, followed by interview process for selection. | Once a year for 2 weeks. | Originally orphans, now villages on the outskirts of Yangon. | The reflections every night became a logistics session instead. Currently planning to do reflections on every meeting. | Approached doctors, especially Burmese doctors for interest in volunteering. Went to Alexandra Hospital for fund-raising. | Originally Myanmar Vision International, currently YMCA. |
| Manila Medical Mission | Overseas, Manila, The Philippines. | Manila Squatter Settlements, North Cemetery, Mangyan tribe on Mindoro Island and the Aeta tribe on Zambales. | To conduct basic medical clinics  To provide medicine  To give education on hygiene, wound dressing,  To provide vaccinations  To conduct evangelism and spiritual outreach that also focuses on the spiritual growth of the team. | 11 medical students: 8 M2s, 3 M1s. | Announcement and publicity followed by a selection process through interview. | Once in six months, 9-12 days each time. | Poor people with low levels of hygiene and insufficient access to water and to just basic needs. Also visit Homes for abandoned children. | Reflection form (Google form) to fill in about spiritual growth. | Sponsorship from Food Empire, sponsorship of stationery from Pilot Pen, oil from Eagle Oil. Personally ask relatives or family and friends for sponsorships, organize small fund raising projects. | Philadelphia Christ Fellowship in Manila. |
| Project Lokun | Overseas, Pursat Province, Cambodia. Recently expanded to Pailin Province. | Pursat and Pailin Province. | To provide house-to-house screening services, conduct a needs assessment, and make referrals to a local healthcare facility for follow-up.  Secondly, to provide health education to target beneficiaries.  Thirdly, to hold clinics for villagers, connect villages to their local healthcare system, bring doctors of some of the rarer specialties like oncologists and ophthalmologists to the villagers. | 25 medical students comprising 15 M2s and 10 M1s | Year 1s fill up an application form to select 2 of the 4 existing Cambodian OCIPs in medical school, and are selected based on individual interviews. | Twice a year, so each trip is about 13-14 days: 10 days of work, 3-4 days of rest and recreation. | Impoverished villagers in Pursat and Pailin who lack basic health education. | Team debrief session every night. After the trip, reflection writing about observations and improvements, useful for the leader to understand things from members’ perspectives. | Donations in kind from organizations like Lee Foundation, donations from Cambodians like Tiger Balm. | Apostolic Prefecture Battambang (CROAP: Centre for Research on Optimal Agricultural Practices), Hand of Hope, Prom Vihear Thor (PVT). |
| Project Khon Kaen | Overseas, Khon Kaen, Thailand. | Inner city of Khon Kaen. | Initially, to provide health education and health screening in inner city of Khon Kaen, as well as orphanages.  Currently, to do health screenings and provide health education to the rural immigrants in the slums of Khon Kaen, and provide medical supplies for wound management  To train health reps in the country and provide supplies they need, such as head-mounted flash lights. | 24 medical students from years 3 to 4. | Open sign-ups, followed by interviews, but accepted everyone. | Once yearly for 10-12 days, Plans to extend it to twice yearly. | 50 families of rural immigrants in the inner city of Khon Kaen. | Group reflection after the trip about possible improvements and direction of the project. Everyone writes out their personal reflection. | Fund-raising within school, donations of money and medical supplies from Lee Foundation, some donations of excess supplies from organizations e.g., Raffles Medical donated vitamins. | None. |
| Payatas Medical Outreach | Overseas, Payatas, Manila, The Philippines. | The largest dump site in Manila, The Philippines. | To improve tuberculosis (TB) screening through the direct observe therapy program, and improve public awareness on TB. The project trained 2 scavengers through a 5-day program to be a DOT officer, which is a WHO approved official treatment for TB  To tackle other needs of the community, such as other medical conditions including hypertensionand skin conditions | Initially began with 10 medical students, now has 5 medical students | Mass email to recruit interested parties. No selection process was involved. | Not specified, 1-2 times a year. | Scavengers. | Spontaneous reflections and discussions for changes to the plan while in Payatas, on tailoring the project to needs of the population. | Sold Pediatric Kits, raised funds through car-washing and other fund-raising methods to pay the DOT officer’s monthly salary of $200. After Acts29 took over, there was no need for fund-raising. | Acts29. |
| Project Battambang | Overseas, Poipet, Phnom Penh, Cambodia. | Initially in Battambang, then moved to Poipet. | To conduct education programmes, like Light A Dream program, where a student is supported from high school to university.  To provide health screenings and follow-ups to the beneficiaries.  To conduct a Teacher Support Program to pay teachers to stay and teach students. | 23 medical students from years 1 and 2. Other volunteers include doctors and nurses. | Year 1s fill up an application form to select 2 of the 4 existing Cambodian OCIPs in medical school, and are selected based on individual interviews. Volunteers were selected based on the following principles, "LIBRAS": Love, Integrity, Beneficence, Respect, Accountability, Sustainability. | Twice a year, December trips and May Scholarship Camps. | 25 High school and University students, 4 or 5 university students. Villagers in Poipet. | Reflections every night during the trip, to talk about values, what happened during the day and sustainability of the project. | Funded from Financial Women’s Association. | Sisters in Poipet, contact point is Mavis. |
| Project Sothea | Overseas, Phnom Penh, Cambodia. | Slums of Phnom Penh in the city’s garbage dump. Battambang, and a rural village next to Kamping Pupui Reservoir. | To provide education on basic hygiene like wound care. To hold clinics and healthcare screenings, and provide acute and symptomatic relief for cough and cold, and vaccinations. To conduct surveys to check for major diseases which need intervention  To bring in doctors with specialized knowledge such as surgeons  To cater to other needs of the beneficiaries such as the need for water filters | 26 medical students | Year 1s fill up an application form to select 2 of the 4 existing Cambodian OCIPs in medical school, and are selected based on individual interviews.  Selection was based on skills possessed, such as marketing, publicity or first-aid. | Once a year for the actual trip, then another time to recce. | People from slums of Phnom Penh who live off scraps, villagers living near Kamping Pupui Reservoir. | Nightly reflections for possible improvements. At the end of the trip, reflections are collected from each member, in email document. | Funded by NUH Pediatrics, volunteer doctors are also from an open call in the NUH Pediatrics department. Asked doctors through emails for donations. Raise money through pledge card of $200. Raise money through t-shirt sales, chocolate sales, | Rotaract Club Singapore, NUH Pediatrics. |

DOT officer: Directly Observed Therapy officer for Tuberculosis; M1: First year medical student in Yong Loo Lin School of Medicine, National University of Singapore; M2: Second year medical student in Yong Loo Lin School of Medicine, National University of Singapore; TB: Tuberculosis.

**Table 4.** Overview of obstacles reported, solutions proposed and how academics were affected as a result of the community service project.

| **Name of community service project** | **Obstacles reported** | **Solutions proposed** | **How academics were affected as a result of the project** |
| --- | --- | --- | --- |
| **Operation Smile Student Chapter** | Difficulty in managing logistics, such as sorting of colours and sizes during the sale of shirts | To form an executive committee for this project, so that there could be potentially better sense of ownership and responsibility in terms of managing logistics | No influence |
|  | Difficulty in managing the volunteers due to lack of structure and absence of an executive committee | To form an executive committee for this project to help manage the volunteers |  |
|  | Low commitment levels of volunteers | To increase the recognition of the project in the school, so that volunteers are more inclined to contribute.  In this case, the school’s help may be necessary in improving the project's image among the medical student population |  |
| **Neighbourhood Health Screening (NHS)** | Difficulty in specific goal setting to improve the project in the future | To create new future ideas for the project with the help of mentors | No influence |
|  | Low commitment levels of volunteers | To ensure that all volunteers contribute to the project, leveraging on the help of mentors |  |
|  | Difficulty in persuading the residents (beneficiaries of the project) to go for follow-up sessions, which is part of the specific aims of this project | To ensure that follow-up for the residents is affordable and convenient.  However, specific methods to do this need to be further planned. |  |
| **Project Happy Apples** | Difficulty in obtaining funds and resources to start the project | To source for potential sponsors through the mentor's contacts | Negative influence, due to the reduced amount of time to study |
|  | Difficulty in specific goal setting on the part of the new committee | To share experiences and lessons learnt with the new committee during handover of the project, so that they are well-equipped to set specific goals and aspirations to improve the project |  |
|  | Difficulty in managing the volunteers, specifically organizing suitable times to interview the palliative patients (beneficiaries of this project) | To restructure the scheduling system by sending any volunteer who is available to interview the palliative patients, to increase flexibility in terms of conducting the interviews |  |
|  | Difficulty in recruiting volunteers, as a large number of volunteers were needed for various aspects of the project | To recruit volunteers outside of medical school when specialized skills were necessary. For example, students from another university (Nanyang Technological University) studying Arts and Design Media were recruited for their design expertise which were required when documenting the stories of the beneficiaries |  |
|  | Difficulty in team management, especially when liaising with people in high positions and dealing with the rules and red tape | To obtain help from the school in terms liaising with organizations, corporations, publicity companies, hospitals and hospices |  |
| **Constructing Care Collaboration (CCC)** | Difficulty in specific goal setting in terms of raising awareness and recognition of the project | To conduct public sharing sessions to raise awareness of the project, and spreading the cause of the project through the media | Positive influence, as the project allowed for revision of clinical medicine leading to a better understanding of academic content |
| **Project Sa’Bai** | Difficulty in terms of time management | To plan ahead and delegate time to do specific tasks | No influence |
|  | Difficulty in managing the volunteers, specifically in terms of ensuring that everyone works at a comfortable pace | To forsee this problem of different paces of volunteers and plan ahead according to each volunteers' pace |  |
|  | Difficulty in obtaining the Singapore fund-raising permit, to raise funds for the project | To obtain help from the school, such as through the school’s Dean’s Office, to obtain the fund-raising permit for the project |  |
|  | Difficulty in empowering the locals, such as in provision of training to the people to improve their healthcare system | Work on the sustainability of the project, set it as a long term goal and work towards it. |  |
|  | Difficulty in recruitment of volunteers, especially doctors as it is difficult for them to be on leave to participate in this project | NIL |  |
| **Project Yangon** | Difficulty in training the beneficiaries to obtain self-sufficiency, as the beneficiaries may not practice what the volunteers have taught them | NIL | No influence |
|  | Difficulty in recruiting and managing volunteers | To obtain help from the school in terms of arranging sharing sessions with the seniors who have gone on multiple OCIP trips and are very knowledgeable, so that insights on planning OCIPs can be shared.  To have a mentor to oversee the project and provide advice in terms of recruitment and managing volunteers |  |
|  | Difficulty due to language barriers | To find and recruit the local doctors as volunteers, so that translators need not be hired |  |
|  | Difficulty in obtaining funding and sponsorship | NIL |  |
| **Manila Medical Mission** | Difficulty in obtaining funds to sustain the project | NIL | Negative influence, due to the reduced amount of time to study |
|  | Difficulty in specific goal setting in terms of raising awareness of the project, such as what exactly to do to ensure objective help to the beneficiaries and ensure results | NIL |  |
|  | Difficulty in addressing safety issues, as the project was conducted in rural and mountainous areas | NIL |  |
| **Project Lokun** | Difficulty in overcoming the language barrier, which was needed to communicate with the Cambodians (beneficiaries of the project), and replying them via social media i.e. a Facebook page | NIL | No influence |
|  | Difficulty in attaining recognition or institutionalizing the project. For example, the official Cambodian Government University (University of Health Sciences) does not allow students to be involved in the project because the project was not institutionalized | To obtain help from the school in terms of publicity or institutionalizing CIPs that are conducted in the faculty, so that overseas volunteers may be involved in the project as well. This may have the positive effect of having the beneficiaries and self-sufficiency to support themselves |  |
|  | Difficulty in obtaining funds and resources to sustain the project | To obtain help from the school in terms of financial support |  |
|  | Difficulty in recruiting volunteers and doctors to provide medical treatment to the beneficiaries | To obtain help from the school, which liaises with NUHS to find doctors to volunteer on the project on the trip  To obtain help in arranging to bring patients from overseas over to Singapore to seek treatment |  |
| **Project Khon Kaen** | Difficulty in obtaining funds and resources to sustain the project | To obtain media coverage, e.g. newspaper coverage, so that there is greater visibility of the project, which may help in terms of obtaining sponsorship | Negative influence, due to the reduced amount of time to study |
|  | Difficulty in funding management, in terms of red tape. For example, the accountability of the funds and whether it was legal under the charity’s acts, and auditing had to be addressed | To obtain help in terms of managing of funds, such as through the help of NUS Medical Society. |  |
|  | Difficulty in obtaining medical equipment such as blood pressure machines and weighing machines | To obtain help from the school in terms of facilitating the borrowing of necessary medical equipment. |  |
|  | Difficulty in management of volunteers to tailor volunteers' time to the needs of the beneficiaries | To obtain help in terms of mentorship, such as in the setting of dates to go every year, planning the optimal number of volunteers recruited.  To obtain advice to understand the beneficiaries and their needs better. For example, to have lessons on the beneficiaries' demographics, common diseases/ viruses and how they spread in the country of the beneficiaries, the screening methods and which diseases should be screened. |  |
| **Payatas Medical Outreach** | Difficulty in adjusting to the culture of the target population, which had a slower pace of life | To plan according to the needs and culture of the community, and understand that change should be gradual | Positive influence, due to an increased sense of purpose and meaning in studying which the project was able to instill |
| **Project Battambang** | Difficulty in obtaining funds and resources to sustain the project | To obtain help from the school in terms of arranging for external organizations to provide funds and resources for this project, for example, by sponsoring necessary drugs.  To obtain a sustainable source of funding. | No influence |
|  | Difficulty in recruiting volunteers, such as doctors and nurses, who would speed up the process of the health screening | To obtain any form of help in terms of recruiting these volunteers |  |
| **Project Sothea** | Difficulty in obtaining funds and resources to sustain the project | To obtain greater publicity for the project, which would help in reaching out to sources of funds and resources | No influence |

CIP: Community Involvement Project. MedSoc: Medical Society (a student society in Yong Loo Lin School of Medicine, National University of Singapore). NUHS: National University Health System.

**Figure 1.** Frequency of common obstacles being cited as a barrier by CIP leaders when they participate in community service project
